# Late Eocene white pines (*Pinus* subgenus *Strobus*) from southern China

**DOI:** 10.1038/srep16390

**Published:** 2015-11-09

**Authors:** Qingqing Xu, Wenjun Zhou, Tatiana M. Kodrul, Serge V. Naugolnykh, Jianhua Jin

**Affiliations:** 1 State Key Laboratory of Biocontrol and Guangdong Provincial Key Laboratory of Plant Resources, School of Life Sciences, Sun Yat-sen University, Guangzhou 510275, China; 2 Department of Paleobiology, National Museum of Natural History, Smithsonian Institution, Washington, DC, 20013, USA; 3 Geological Institute, Russian Academy of Sciences, Moscow 119017, Russia

## Abstract

Fossil records indicate that the genus *Pinus* L. split into two subgenera by the Late Cretaceous, although subgenus *Strobus* (D. Don) Lemmon is less well documented than subgenus *Pinus* L., especially in eastern Asia. In this paper, *Pinus maomingensis* sp. nov. is established based on a compressed seed cone from the upper Eocene of the Maoming Basin of southern China. This species is attributed to genus *Pinus*, subgenus *Strobus*, section *Quinquefoliae* Duhamel, subsection *Strobus* Loudon based on the combination of morphological characters obtained from the cone scales, specifically from the terminal umbo, rhombic apophysis, and cuticle structure. Associated fascicles of needle leaves with deciduous sheaths and bulbous bases are recognized as *Pinus* sp. and also represent *Pinus* subgenus *Strobus*. This new discovery from the Maoming Basin constitutes the first megafossil record of subgenus *Strobus* from southern China and implies that the members of this subgenus arrived in the southern region of China by the late Eocene. The extant species of subgenus *Strobus* are mainly distributed in northern temperate and tropical to subtropical mountainous regions. We propose that the Maoming Basin was adjacent to a mountainous region during the late Eocene.

*Pinus* L., consisting of more than 110 extant species, is the largest and the most widespread genus of Pinaceae in the Northern Hemisphere^1^,^2^,^3^. This genus is subdivided into subgenus *Pinus* L. (subgenus *Diploxylon* (Koehne) Pilger, the hard pines) and subgenus *Strobus* (D. Don) Lemmon (subgenus *Haploxylon* (Koehne) Rehder, the white or soft pines)^3^,^4^,^5^,^6^. The major differences between the above two subgenera are the number of fibrovascular bundles per needle, sheath persistence and the position of the umbo on ovuliferous scales. Their general distinguishing criteria are listed in Table 1.

The origin of the genus *Pinus* is thought to date to the Early Cretaceous^7^. Ryberg *et al.*^8^ suggest that some species of the fossil genus, *Pityostrobus*, might be reassigned to genus *Pinus* and the evolutionary diversification of Pinaceae began earlier than previously recognized from fossil evidence. Currently the oldest fossil record of this genus is a seed cone *P. yorkshirensis* Ryberg, Stockey, Hilton, Mapes, Riding et Rothwell^8^. It was discovered in the Early Cretaceous Wealden Formation (Fm.) of Yorkshire, United Kingdom, and was placed in subgenus *Pinus* based on morphological and anatomical structure. Although pine fossils are well documented in a variety of stratigraphic and geographic settings^9^, fossil records of the two subgenera of *Pinus* differ greatly in their past abundances. Most of these fossils have affinity to subgenus *Pinus* and relatively few have been confidently placed in subgenus *Strobus*^2^,^10^. Cretaceous fossil needles of *Pinus* sp.^11^, *Pinus yezoensis*^12^, and a fossil cone of *Pinus magothensis*^13^ were initially accepted as members of subgenus *Strobus*. However, they were later thought to have affinity with subgenus *Pinus* or possibly with other pinaceous genera^14^,^15^. Presently, the rise of subgenus *Strobus* can be dated confidently to the permineralized wood, *Pinuxylon* sp., from the Late Cretaceous Aachen Fm. of northeastern Belgium^16^. More recent records of subgenus *Strobus*, for example ovulate cones, occur in the mid-Eocene strata^10^, no megafossils are known from the Paleocene^7^.

The megafossil record of subgenus *Strobus* in China is very restricted. Only two Pliocene fossil wood species from Yunnan Province, *Pinus armandii* Franchet and *P.* cf. *armandii*, were reported by Yi *et al.*^17^,^18^. In this study, we describe two species of subgenus *Strobus* based on a fossil pine cone and needle remains collected from the Huangniuling Fm. of the Maoming Basin in southern China (21°42′ N, 110°53′E) (Fig. 1).

The Maoming Basin is a small, upper Mesozoic to Cenozoic sedimentary basin oriented along a northwestern to southeastern axis in Guangdong Province. The Upper Cretaceous to Neogene deposits of the Maoming Basin are subdivided into eight formations; in ascending order they are: the Sanyajiang Fm., Tongguling Fm., Shangdong Fm., Youganwo Fm., Huangniuling Fm., Shangcun Fm., Laohuling Fm., and Gaopengling Fm^19^,^20^. The Huangniuling Fm., from which the megafossils were recovered, consists principally of fluvial grey, yellow to white sandstones, siltstones and conglomerates with beds and lenses of varicolored mudstones and claystones, ranging from whitish pink to grayish green to brownish gray. Based on a magnetostratigraphic study by Wang *et al.*^21^, buttressed by palynological data by Aleksandrova *et al.*^22^, the age of the Huangniuling Fm. is considered to be late Eocene. Therefore, the occurrences of fossil pine cone and needle remains from Huangniuling Fm. provide new information on the distribution of subgenus *Strobus* and imply that the members of this subgenus arrived in the southern region of China by the late Eocene.

## Results

**Systematics**.

**The fossil cone.**

Family: Pinaceae Lindley, 1836

Genus: *Pinus* Linnaeus, 1753

Subgenus: *Strobus* (D. Don) Lemmon, 1983

Section: *Quinquefoliae* Duhamel, 1755

Subsection: *Strobus* Loudon, 1838

Species: *Pinus maomingensis* Xu, Jin, Zhou, Kodrul et Naugolnykh sp. nov.

### Etymology

The species name is derived from the Maoming Basin where the specimens were collected.

### Holotype

MMJ2-1-005a (Fig. 2A) and MMJ2-1-005b (Fig. 2B), part and counterpart of a seed cone, which has seeds inside.

### Paratype

MMJ2-1-006 (Fig. 2E), an isolated ovuliferous scale.

### Repository

The Museum of Biology of Sun Yat-sen University, Guangzhou, Guangdong Province, China.

### Type locality and horizon

**Figure 1:**
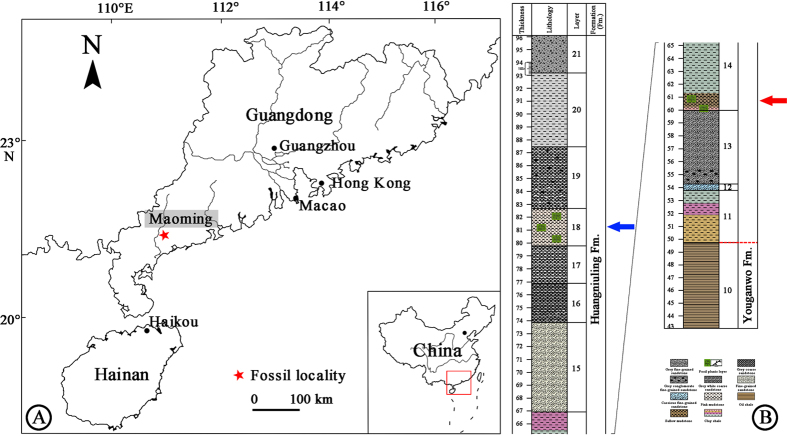
Geographic map of the Maoming Basin, Guangdong Province, China and stratigraphic column of the fossil locality. (**A**) location of the Maoming Basin (red star) (drawn by Q.X., using Adobe Photoshop CS5). (**B**) Stratigraphic column of Huangniuling Formation, modified from Aleksandrova *et al.*^22^. The fossil cone and the single isolated ovuliferous scale were collected from layer 14 (red arrow), and the needle fossils were collected from layer 18 (blue arrow).

Huangniuling Fm., upper Eocene. Specimens were collected in Jintang Town, Maoming City, Guangdong Province (Fig. 1).

### Diagnosis

Seed cone oblong-elliptical to cylindrical. Ovuliferous scales thin with longitudinal ridges on abaxial side, spirally arranged. Apophyses rhomboidal to broadly rhomboidal; no sealing band. Umbo terminal, slightly sunken, bearing a slightly swelling transverse ridge in a diamond-shaped area.

### Description

**Figure 2:**
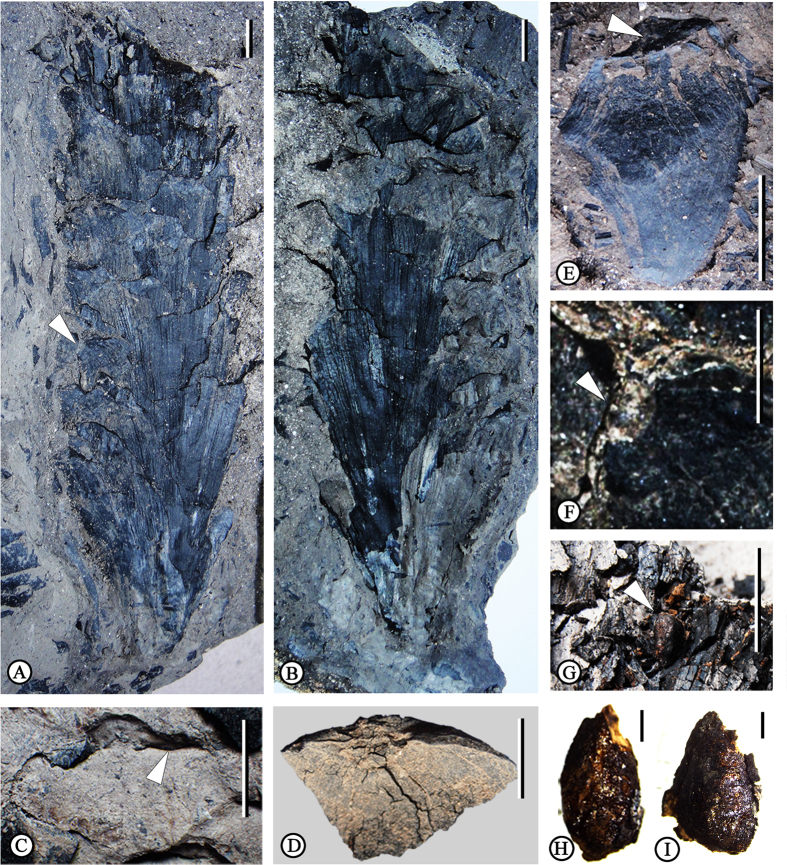
*Pinus maomingensis* sp. nov. (**A,B**) Holotype; part (**A**, MMJ2-1-005a) and counterpart (**B**, MMJ2-1-005b) of the cone with ovuliferous scales, arrowhead in (**A**) points to the protrusion. (**C**) Rhombic apophysis from (**B**) arrowhead points to the reflexed ridge. (**D**) Upper part of apophysis from (**B**) showing details of terminal umbo. (**E**) Paratype (MMJ2-1-006); single ovuliferous scale, arrowhead points to the protrusion. (**F**) The protrusion (arrowhead) in (**A**). (**G–I**) Seeds show little seed coat, arrowhead in figure (**G**) shows the position of the seed in the cone. Scale bars: **A**–**C**,**E**,**G** = 1 cm; **D**,**F**,**H**,**I** = 0.5 cm.

The seed cone (Fig. 2A,B) is variably oblong to elliptical or cylindrical. The visible portion of the seed cone is 16 cm in length. The cone width, generally consistent over the middle and upper part of the cone, is ca. 5 cm. The basal part of the cone is more or less cuneate (probably due to incomplete preservation) and ca. 1 cm in diameter. The ovuliferous scales are thin, oblong-obovate, helically arranged around the axis, about 5 cm long, 2.4 cm wide, and about 0.1 cm thick in the middle, with a slightly expanded apophysis (Fig. 2D). The isolated ovuliferous scale (Fig. 2E) is rhomboidal to obovate, sessile, ca. 2.6 cm long by 2 cm wide. Apophyses are rhomboidal to broadly rhombic. Longitudinal ridges and furrows are clearly visible on the abaxial side of the scales. The umbones (Fig. 2A,D,E) are terminal and slightly sunken. A small protuberance (Fig. 2A,F) occurs in the umbonal area, also present in the isolated ovuliferous scale (Fig. 2E). A pair of arched ridges (Fig. 2C,D) near the umbo stretches transversely, gradually becomes robust and slightly recurved, then merges with upper lateral margin of the apophysis. Seeds (Fig. 2H,I) are elliptical to fusiform in shape, ca. 6 mm long by 3 mm wide.

**Figure 3:**
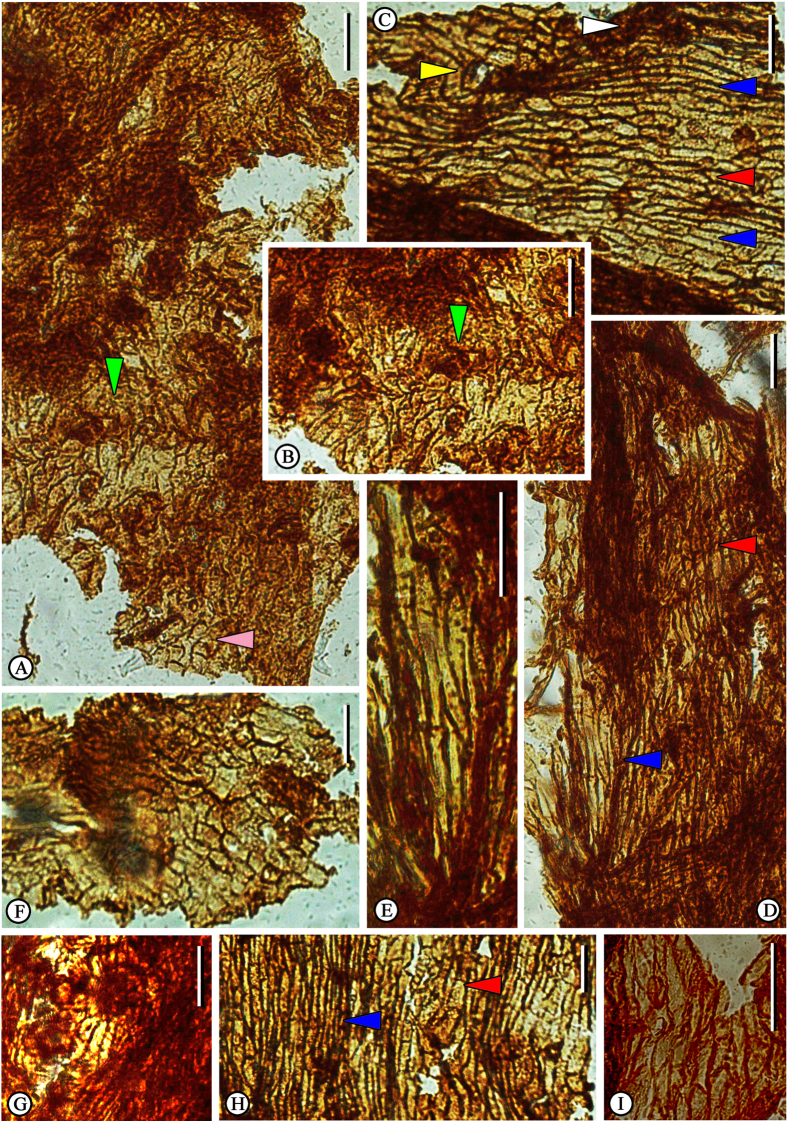
Cuticle of *Pinus maomingensis* sp. nov. obtained from the apophysis. (**A**) The cells from the ridge area (green arrowhead); pink arrowhead shows the nearby irregularly shaped cells. (**B**) Enlarged from (**A**), the green arrowhead shows the ridge. (**C,D**) The longitudinally elongate cells (blue arrowhead) and irregularly polygonal cells (red arrowhead) from the ribs and furrows, yellow arrowhead in (**C**) shows the unicellular trichome, and white arrowhead in (**C**) shows the trichome base. (**E**) The longitudinally elongate cells, enlarged from (**D**). (**F**) The irregularly shaped cells. (**G**) Trichome base. (**H**) Cuticle shows the longitudinally elongate cells (blue arrowhead) and irregularly polygonal cells (red arrowhead). (**I**) Irregularly polygonal cells. Scale bar = 50 μm.

Three types of epidermal cells (Fig. 3) were retrieved from the upper part of the abaxial surface of ovuliferous scale: (i) longitudinally elongate cells (Fig. 3C–E,H); (ii) irregular polygonal cells (Fig. 3C,D,H,I); and (iii) irregularly shaped cells (Fig. 3A,F). Anticlinal walls of all epidermal cells are well developed and express slightly undulatory outlines. In longitudinally elongate cells, the end walls are either transverse or oblique to the side walls. The trichome bases (Fig. 3C,G) are composed of two or three rings of small rectangular epidermal cells, and unicellular trichomes (Fig. 3C) are sparsely dispersed on these cuticles. No stomatal complexes were observed.

**The fossil needles.**

Family: Pinaceae Lindley, 1836

Genus: *Pinus* Linnaeus, 1753

Subgenus: *Strobus* (D. Don) Lemmon, 1983

Species: *Pinus* sp.

### Referred specimens

MMJ3-002a, MMJ3-002b, MMJ3-003, MMJ3-037 to MMJ3-049, MMJ3-094 to MMJ3-097, fossil needles.

### Repository

The Museum of Biology of Sun Yat-sen University, Guangzhou, Guangdong Province, China.

### Type locality and horizon

Huangniuling Fm., upper Eocene. Specimens were collected in Jintang Town, Maoming City, Guangdong Province (Fig. 1).

### Description

**Figure 4:**
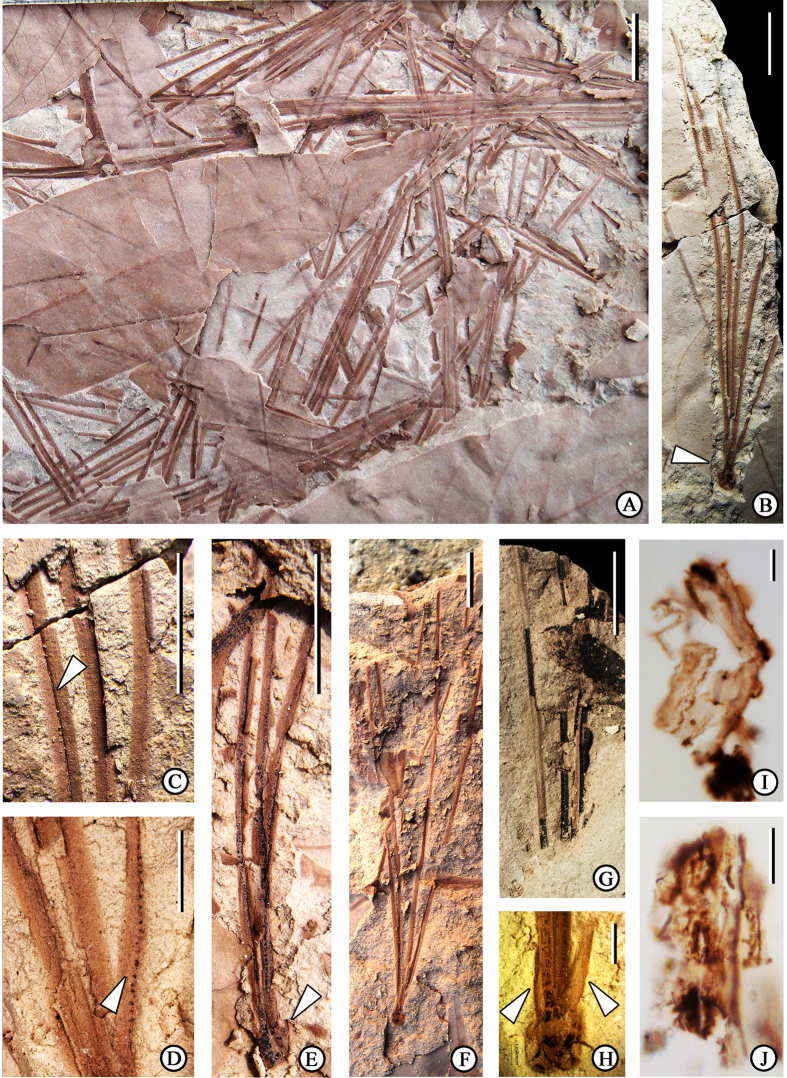
Leaves of *Pinus* sp. (**A**) Showing the preservation of needles (MMJ3-094). (**B**) Fascicle bearing five needles, showing bulbous base (arrowhead) (MMJ3-038). (**C**) Longitudinal furrow (arrowheads) of the needle, enlarged from (**B**). (**D**) Finely serrate margins (arrowheads), enlarged from (**B**). (**E**) Fascicle bearing three needles, showing bracts (arrowhead) and leaf scars (MMJ3-095). (**F**) Needle fascicle bears four needles (MMJ3-096). (**G**) Fascicle from which cuticle fragments were obtained bears four needles (MMJ3-002-a). (**H**) Bulbous base (MMJ3-097), arrowhead points to a pair of sheath scales. (**I,J**) Cuticle fragments obtained from (**G**), showing epidermal cells and stomatal complexes. Scale bar: **A**,**B**,**E**–**G** = 1 cm; **C** = 0.5 cm; **D**,**H** = 0.2 cm; **I**,**J** = 50 μm.

The short shoot leaves are in fascicles with deciduous basal sheaths. The number of needles per fascicle is mostly five but varies from 3 to 5 (Fig. 4A–G). The needles are up to 10 cm long by ca. 1 mm wide, with triangular in cross section (Fig. 4C) and finely serrate margins (Fig. 4D). Needle widths are uniform along their entire length. The fascicles have bulbous bases (Fig. 4B,E,F,H). Membranous sheaths are deciduous at maturity, but they are retained (Fig. 4H) in the young fascicles.

Cuticles obtained from fossil needles preserve the following characters: Epidermal cells are rectangular and longitudinally oriented (Fig. 4I). The anticlinal walls of epidermal cells are straight or sinuous and slightly thickened. Stomatal complexes are paracytic, elliptical to oval in shape (Fig. 4J), and arranged in longitudinal rows. Well-developed cuticular flanges are preserved between guard cells and subsidiary cells. The polar subsidiary cells are smaller than the lateral subsidiary cells.

## Discussion

### Classification of the species with terminally-positioned umbones

Over 40 taxonomic treatments have been proposed for the genus *Pinus*^4^,^23^,^24^. The early classificatory systems are based mainly on morphology^25^,^26^, whereas more recent studies use molecular phylogenetic approaches^4^,^6^,^23^,^27^. The recent system of *Pinus* by Gernandt *et al.*^6^ based on rbcL + matK gene sequences and morphological characters is relatively widely accepted. This classification includes two subgenera (*Pinus* and *Strobus*), four sections (*Pinus*, *Trifoliae* Duhamel, *Quinquefoliae*, *Parrya* Mayr) and 12 subsections. The strictly North American section *Parrya* is restricted to the subsections *Cembroides* Engelmann, *Nelsoniae* Van Der Burgh, and *Balfourianae* Engelmann and is characterized by a dorsal umbo on the ovulate cone scale. The subsections *Strobus*, *Krempfianae* Little et Critchfield, and *Gerardianae* Loudon have been placed in the Eurasian and North American section *Quinquefoliae*. The group with a terminally-positioned umbo on the ovulate cone scale was regarded as one subsection (subsection *Strobus*) of section *Quinquefoliae* instead of subdividing them into subsection *Strobus* (the correct name for subsection *Strobi*^6^) and *Cembrae* Loudon^4^,^23^,^26^,^27^. In our study, the classification of Gernandt *et al.*^6^ is adopted.

### The fossil cone

**Figure 5:**
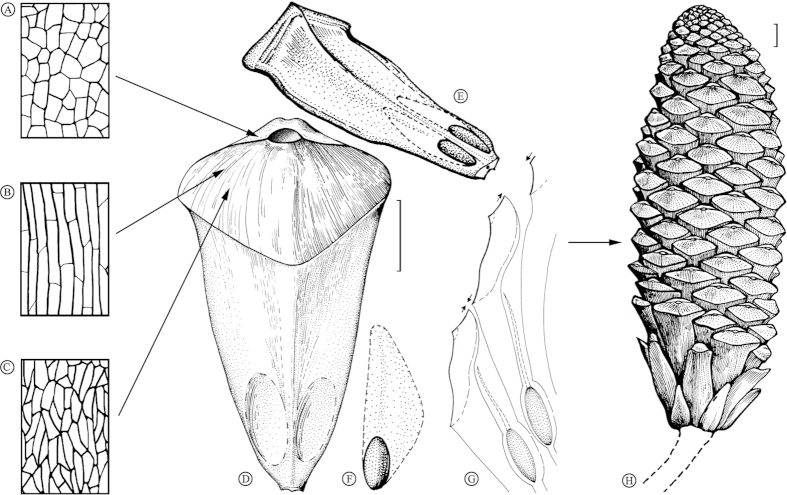
Reconstruction of *Pinus maomingensis* sp. nov. (**A–C**) Three kinds of epidermal cells of *Pinus maomingensis* sp. nov.: (**A**) shows irregularly shaped cells, (**B**) shows the longitudinally elongate cells, and (**C**) shows irregularly polygonal cells. Sketched from Fig. 3C,F. Arrows in the right show their positions in the ovuliferous scale. (**D**) Lower side of an ovuliferous scale. Its entire length was measured from the middle ovuliferous scales of Fig. 2A, and width and shape of the apophysis was derived from the imprints of Fig. 2B,C,E. Details of umbo area derived from Fig. 2B,D,E. Scale bar = 1 cm. (**E**) Upper side of an ovuliferous scale sketched from a side view. (**F**) Seed which may have a wing. (**G**) Part of a longitudinal section of the cone. (**H**) General reconstruction of the cone with ovuliferous scales helically arranged around the axis. (**A–H**) drawn by Q.X. and S.N. Scale bar = 1 cm.

The fossil cone (Fig. 2 and Fig. 5) described above possesses the following morphological characters: (i), thin ovuliferous scales helically arranged around the axis; (ii), the apex of the ovuliferous scale is slightly inflated with an apophysis and terminal umbo. Consequently, this fossil cone can be assigned to the genus *Pinus*, according to distinguishing features summarized by Miller^9^. The umbo position and absence of a sealing band on the lower side of apophysis exclude a close affinity to the subgenus *Pinus*^28^, and judging from its distinct terminal umbo, this cone can be easily classified into the subgenus *Strobus*. The isolated ovuliferous fossil scale is considered to be conspecific with the cone due to the morphological similarities of the apophysis and umbo. Wing development and the size of seeds also are important in *Pinus* classification^5^. However, seeds discovered in the middle and upper part of the present cone provide only limited evidence for classification, principally because it is difficult to distinguish whether these seeds are winged or not.

### Comparisons with fossil cone taxa

*Pinus* existed at the onset of the Cretaceous and is well documented for the Cretaceous and Cenozoic^29^. Relatively few Cretaceous fossils have affinity with the subgenus *Strobus* and some specimens originally attributed to this subgenus were reassigned to the subgenus *Pinus* or other genera. *Pinus magothensis* Penny, from the Magothy Fm. of Delaware, U.S.A. is 9–10 cm long by 3–4 cm wide with thin flattened scales, each subtending two winged seeds; and with a thin apophysis and an inconspicuous terminal umbo^13^. There is no detailed description for the apophysis and umbo of *P. magothensis*, therefore it is difficult to compare it with *P. maomingensis*. *Pinus magothensis* was considered to be the most important evidence for the subgenus *Strobus* during the Cretaceous. However, Miller^29^ thought the characters were insufficient to show conclusive affinity with this subgenus due to the lack of details involving internal structure. As a result, the cone was reassigned to *Pityostrobus* by Miller and Malinky^14^. Willyard *et al.*^15^ also accepted this reassignment.

The fossil record of pines continues into the Paleogene and Neogene where fossil seed cones are more abundant and are overwhelmingly assigned to the subgenus *Pinus*^10^,^14^. The diversity of cone types, as well as the megafossil record of external impressions, implies that a number of species in various subsections were already in existence during the Eocene^10^. Those cones, such as *Pinus lindgrenii* Knowlton^30^, *P. balfouroides* Axelrod^31^ and *P. sanjuanensis* Axelrod^10^, appear assignable to the subsections of section *Parrya* and are excluded for comparison here. We focus on those fossil cones that exhibit affinity to the section *Quinquefoliae*, subsection *Strobus* sensu Gernandt *et al.*^6^.

The middle Eocene cone *Pinus delmarensis* Axelrod^10^, from the Del Mar Fm. near San Diego, California (U.S.A.) is larger (estimated as 26 cm long) than *P. maomingensis*, but is the most similar fossil species to our specimens. The similarities involve overall shape and the size of the ovuliferous scale and terminally positioned umbo. However, the lack of detailed published information on the apophysis and umbo of *P. delmarensis* precludes an informative comparison. Axelrod^10^ observed that *P. delmarensis* was very similar to the extant species *P. lambertiana* Douglas. However, the extant species *P. lambertiana* is readily distinguished from *P. maomingensis* (see the comparison with extant species below), indicating that additional detailed comparisons with *P. delmarensis* are unnecessary.

*Pinus florissanti* Lesquereux^32^ was established based on an ovoid seed cone with large scales from the upper Eocene Florissant Fm., Colorado, subsequently supplemented with seeds and needles by MacGinitie^33^. MacGinitie^33^ also reassigned *P. sturgisi* Cockerell^34^ to this species. *Pinus florissanti* was considered to be related to extant *P. ponderosa* Douglas ex C. Lawson (subgenus *Pinus*)^32^,^33^, but Axelord^10^ suggested that this fossil species was more closely allied to *P. flexilis* E. James (subgenus *Strobus*, section *Quinquefoliae*, subsection *Strobus*) because the characters of the cone and needles of *P. florissanti* were similar to those of the extant *P. flexilis*. Millar^7^ also supported the close affinity of *P. florissanti* with subsection *Strobus*. The cone of *P. florissanti* is shorter but wider (11 cm long by 6 cm wide^33^) than our specimens, and the cone scales are shorter and narrower (4.5 cm long by 1.5 cm wide^33^). Furthermore, this fossil species differs from our specimens in having conical and rhomboidal umbo.

*Pinus echinostrobus* Saporta^35^ from the upper Oligocene of Armissan in Aude, France, has an ovate, flat and slightly striated apophysis with a terminal umbo. The nearest living relative of this species was considered to be *P. koraiensis* Siebold et Zuccarini^36^ which is distinguished from our specimen by having a reflexed apex with a basal-uncinate umbo. The cone of *P. echinostrobus* differs from our specimen in that the cone scales are shorter (1.0–1.4 cm long by 1.2–1.4 cm wide) and the apophysis is slightly curved and basally uncinate at the end of the umbo.

*Pinus grossana* Ludwig^37^ was recovered from the lower Miocene of Rockenberg locality of Wetterau, Germany. This cone is similar in width (5.5 cm) but is much longer (23 cm) than the present specimen according to the descriptions of Mai^36^. The thin cone scale with stripes on the abaxial side and the rhomboidal and slightly convex apophysis with a slightly reflexed apex are both similar to our specimens, although *P. grossana* is much wider and its length is unknown. In addition, this species differs from *P. maomingensis* in its erect, conical umbo. Ludwig^37^ thought that the nearest relative of this species was *P. lambertiana*. However, Mai^36^ considered that the overall features of the cone and the structure and testa anatomy of its associated winged seed indicated a relationship with extant *P. wallichiana* Jackson. This extant species is different from *P. maomingensis* in having wedge-shaped cone scales and grooved apophyses with an obviously incurved apex and a blunt umbo.

*Pinus letzii* Kirchheimer^38^ was described from the upper Miocene of the lower Rhenish Basin, Germany. The cones were 6–12 cm long by 2.5–3.5 cm wide and the cone scales were 2.5–3 cm long by 1–1.7 cm wide. The closest relative of *P. letzii* is thought to be *P. dalatensis* de Ferré^36^,^39^. The cone of *P. letzii* is shorter and narrower than that of *P. maomingensis*. Additionally, the flat apophyses of *P. letzii* are triangular or pentagonal in shape, with a triangular and terminal sunken umbo, whereas *P. maomingensis* bears rhombic to broadly rhombic apophyses with a diamond shaped and sunken umbo.

*Pinus monticola* var. *fossilis*^40^ from the Pliocene of Siberia, Russia, differs from our present specimens in possessing more robust and thicker cone scales. This species was once thought to be similar to the extant American species *P. monticola* Douglas ex D. Don^41^, but Axelrod^10^ assessed its affinities as more nearly allied to extant Asian species, notably *P. armandii* Franchet of central China. The extant species *P. armandii* differs from *P. maomingensis* in having a triangular or rhombic and thickened apophysis with an obtuse umbo. The Miocene cone *P. itelmenorum* Dorofeev^42^ from the Mammoth Mountain flora of Aldan River, Russia, is similar to *P. monticola* var. *fossilis*. However, this relatively complete fossil cone bears more massive and broader, thicker scales. *Pinus itelmenorum* also shows a relationship with the extant species *P. armandii* and its relatives, and this fossil species can also be distinguished from our specimens.

### Comparisons with extant cone taxa

Almost all members of subsection *Strobus*^6^ have been compared with *Pinus maomingensis*. The results show that all members of subsection *Cembrae* sensu Price *et al.*^4^ are easily distinguished from *P. maomingensis* because they bear an erect or basal-uncinate umbo. Other species, in the subsection *Strobus* sensu Price *et al.*^4^, including *P. ayacahuite* Ehrenberg ex Schlechtendal, *P. flexilis* James, and *P. lambertiana* that bear an apical-uncinate or basal-uncinate umbo, are excluded. The other nine species which possess a similar apophysis shape and an obscure, terminal umbo are listed in Table 2 for detailed comparison. *Pinus dalatensis* and *P. morrisonicola* Hayata show less similarity with our specimens in having smaller cone scales with recurved or slightly recurved terminal umbones. Additionally, *P. morrisonicola* bears an obtuse apophysis apex. *Pinus dabeshanensis* Cheng and Law has a weakly developed umbo, but the apex of the apophysis is obtuse and the upper lateral side of the apophysis clearly is reflexed. The umbones of *P. chiapensis* (Martínez) Andresen, *P. peuce* Grisebrach, and *P. kwangtungensis* Chen are flat, straight, or slightly incurved, and this character is different from that of *P. maomingensis*. The species *P. fenzeliana* Handel-Mazzetti, *P. parviflora* Siebold et Zuccarini, and *P. wangii* Hu et Cheng bear sunken to slightly sunken umbones which are very similar to the present specimens. However, the apex of apophysis of *P. parviflora* is rounded and its lateral side is reflexed. The lateral side of the apophysis of *P. wangii* is slightly incurved.

After detailed comparisons with the extant species, the results show that, although the cones of *Pinus fenzeliana* are somewhat smaller than the cone of *P. maomingensis*, it is the most similar extant species given its cone’s general shape, possession of both a broadly rhombic apophysis with slightly reflexed upper lateral side and a sunken umbo. Nevertheless, there is a subtle difference in the umbonal area. The middle part of the umbonal area of *P. maomingensis* is sunken and slightly folded, and the transversely stretched ridge is situated near the center of the umbonal area, and extends to the slightly reflexed apex. This character seems more similar to the cone scale apex of *P. sibirica* Du Tour of the subsection *Cembrae* sensu Price *et al.*^4^, but a key difference is that *P. sibirica* has a basal-uncinate umbo. Although we find two slight protrusions on the center umbonal area of our specimens, there is insufficient evidence to conclude that the fossil cone is a member of the subsection *Cembrae* sensu Price *et al.*^4^. *Pinus maomingensis* illustrates the close relationship between the subsections *Strobus* and *Cembrae* of Price *et al.*^4^ which were combined into one subsection (subsection *Strobus*) by Gernandt *et al.*^6^.

The cone morphology and epidermal structure of the cone scales of *Pinus maomingensis* was compared with modern species and was found to have similarities both to *P. armandii* and *P. fenzeliana*. *Pinus armandii* is similar to our specimens in the general size and shape of its cone and cone scales. However, epidermal structures of the fossil cone are more similar to those of *P. fenzeliana*. Both of these species possess three kinds of epidermal cells: longitudinally elongate cells, irregular polygonal cells, and irregularly shaped cells. They also share longitudinally elongate cells and irregularly polygonal cells over the ribs and furrows. However, *P. fenzeliana* has longer cells over the ribs. The trichome bases of *P. fenzeliana* have one ring of epidermal cells, and the shapes of these cells are different from those of the fossil cone. *Pinus armandii* has shorter epidermal cells over the ribs, and the trichomes have two rings of epidermal cells surrounding their bases. However, they are distinguished from *P. maomingensis* by smaller numbers and the trapezoidal shape of epidermal cells.

Therefore, based on the detailed comparisons, we conclude that our specimens represent a new species of subgenus *Strobus*, section *Quinquefoliae*, subsection *Strobus*. We formally describe it as *Pinus maomingensis* sp. nov., in light of the following defining characters: (i) the shape and size of the cone; (ii) the shape and size of the cone scales; (iii) the shape, size and umbo of apophyses; (iv) epidermal structures.

### The fossil needles

Abundant fossil needles of pinaceous affinity were collected from the Huangniuling Fm. of the Maoming Basin. A majority of the specimens have a bulbous base and deciduous sheaths. These characters also are diagnostic of needle fascicles of the subgenus *Strobus*^3^,^4^,^5^. Because scales of the fascicle sheaths abscise as the needles elongate in most species of this subgenus^4^, we speculate that our specimens were preserved in different growth stages, as we found a pair of sheath scales arising from surrounding bud scales that had not yet abscised. The bracts that subtend the fascicles are non-decurrent in subgenus *Strobus*^4^,^5^,^28^; this feature is clearly displayed in several fossil specimens. Moreover, characters of well-preserved fossil fascicles are very similar to subgenus *Strobus* needle fascicles described by Stults *et al.*^43^ from a Pliocene deposit in the coastal plain of the Gulf of Mexico. Both of these taxa have short, bulbous fascicle bases. Both species have deciduous sheaths. Consequently, we believe that the fossil needle fascicles associated with the same sediments as *P. maomingensis* also belong to subgenus *Strobus*.

The number of needles per fascicle is almost constant within many species of pines, and frequently has been used as a species-specific character in many taxa^2^,^4^. Among the *Haploxylon* pines, the number of needles per fascicle always is five in section *Quinquefoliae*, but ranges from one to five in section *Parrya*. Fossil needle fascicles collected from the Huangniuling Fm. of the Maoming Basin mostly consist of five needles, although several specimens are preserved as three or four needles, possibly due to the preservation conditions of fossils or needles that fell singularly from mature fascicles with deciduous sheaths^44^. The margins of fossil needles are finely serrate. The needles of the extant species in the same section *Quinquefoliae* are finely serrate in eastern Asia and entire in North America^10^. Species of section *Parrya* that grow in China also possess finely serrate needles.

The anatomical characters of needles, such as the number of vascular bundles, the number and position of resin canals, and cuticular structure also provide important characters for *Pinus* classification^45^,^46^,^47^,^48^. Although a large number of fossil needles were collected from the Huangniuling Fm. in the Maoming Basin, most are preserved as impressions, and only a few specimens are preserved replete with cuticle fragments which show features of epidermal cells and stomatal complexes. Since cuticle characters of fossil needles were incomplete and lack anatomical structure in cross section, it is unreasonable to refer them to a certain species. These fossils are recognized as *Pinus* sp.

### Biogeographic implications

The fossil record indicates that genus *Pinus* split into two subgenera by the Late Cretaceous. The earliest definitive representative of the subgenus *Strobus* was discovered in the Late Cretaceous (Santonian) Aachen Fm. of northeast Belgium^16^. During the Paleogene, the subgenus *Strobus* was common in Eurasia and North America^10^,^36^,^49^ but very rare in China. *Pinus maomingensis* sp. nov. and *Pinus* sp., collected from the Huangniuling Fm. in the Maoming Basin of Guangdong Province, assuredly belongs to subgenus *Strobus*. The discovery of these fossils indicates that white pines were distributed in southern China by at least the late Eocene.

Palynological assemblages from the Huangniuling Fm. of the Maoming Basin of Guangdong suggest that the late Eocene was warm and humid^22^. In addition, many fossil plants collected in the Maoming Basin, such as Podocarpaceae, Arecaceae, Dipterocarpaceae, Annonaceae, Juglandaceae, Euphorbiaceae, Myrtaceae, Fagaceae, Altingiaceae, and Lauraceae^22^, provide evidence for tropical-subtropical components in the Eocene Maoming flora. The subgenus *Strobus* mainly inhabits the north and tropical-subtropical mountainous regions of China and grows well in habitats characterized by temperate and moist climate^5^,^50^,^51^,^52^. Presently, however, white pines do not occur in or adjacent to the Maoming Basin. Currently, only two species—*Pinus fenzeliana* and *P. kwangtungensis*—are naturally distributed in mid to high altitude areas of southern China. These two species grow at an altitude of ca. 1000 m in the northern part of Guangdong Province (Lechang and Ruyuan Mountains) and the Wuzhi Mountains of Hainan Island^5^. Because of these distributional patterns, we propose that the Maoming Basin was adjacent to a mountainous region during the late Eocene.

## Methods

A seed cone, one isolated ovuliferous scale and large numbers of needles were collected from the Huangniuling Fm. of the Maoming Basin, southern China. This cone and the isolated ovuliferous scale are preserved as compressions. Cuticular fragments were obtained from the distal part of the ovuliferous scales of the compressed cone (MMJ2-1-005b). Most needles are impressions, and only a few needle fragments preserved, albeit poorly, epidermal remains. Leaf cuticular fragments were obtained from specimen MMJ3-002a. Specimens were photographed using a Canon EOS 500D digital camera. Photomicrography of the seeds was done with using a Leica S8ap0 and Image-Pro software. The terminology for morphological description of subgenus *Strobus* follows Klaus^53^, Fu *et al.*^5^ and Earle^3^.

Fossil cuticle pieces were removed from the specimens and immersed in 30% HNO_3_ solution overnight, and then washed in distilled water 2 or 3 times. The samples then were treated with very weak (ca. 1–2%) ammonia for 4 hours, and then washed with distilled water. Cuticles were mounted on glass slides and observed and photographed using a Nikon microscope in transmitted light and processed with Adobe Photoshop CS5 (Adobe Inc., San Jose, California, USA). All the fossil specimens and slides are stored at the Museum of Biology of Sun Yat-sen University, in Guangzhou, China.

## Additional Information

**How to cite this article**: Xu, Q. *et al.* Late Eocene white pines (*Pinus* subgenus *Strobus*) from southern China. *Sci. Rep.* **5**, 16390; doi: 10.1038/srep16390 (2015).

## Figures and Tables

**Table 1:** General distinguishing criteria between subgenus *Pinus* and subgenus *Strobus* (data come from Richardson & Rundel^2^; Earle^3^).

Subgenus	Fascicle				Ovuliferous scales		
	Needle number	Fibrovascular bundle	Sheath	Pulvini	Sealing band	Umbo	Seed wing adnation
*Pinus*	2–6	2	persistent	decurrent	yes	dorsal with a spine or prickle	no
*Strobus*	1–5	1	deciduous	non-decurrent	no	terminal without prickles in section *Quinquefoliae*; dorsal with prickles in section *Parrya*	yes

**Table 2:** Comparisons of *Pinus maomingensis* sp. nov. with terminal umbo in subsect. *Strobus* sensu Price *et al.* 4.

Species	Cone		Ovuliferous scale in middle		Apophyses in middle		Umbo	Reference
	Shape	Size (cm)	Shape	Size (cm)	Shape	Apex & Lateral		
*P. maomingensis* sp. nov.	oblong-elliptical to cylindrical	16.0 × 5.0	oblong-obovate, rhombic-obovate	5.0 × 2.4	rhombic to broadly rhombic	flat, slightly thickened; slightly reflexed	slightly sunken; two protruded	Present study
*P. fenzeliana*	narrowly ovoid, or ellipsoidal-ovoid	6.0–14.0 × 3.0–6.0	cuneate oblong- obovoid	2.0–2.5 × 1.5–2.	broadly sub- rhombic	thickened; obviously reflexed	slight sunken; strongly reflexed	Fu *et al.*^5^
*P. dabeshanensis*	cylindrical-ellipsoid	ca.14.0 × 4.5–8.0	oblong-obovoid	3.0–4.0 × 2.0–2.5	rhombic	obtuse, thin; obviously reflexed	not obvious	Fu *et al.*^5^
*P. dalatensis*	cylindrical, straight or crescent shaped	6.0–23.0 × 2.0–9.0	cuneate-elliptical	ca.3.0 × ca.1.5	oblong-rhombic	non-recurved or slightly recurved	slightly recurved	Earle^3^
*P. parviflora*	ovoid or ovoid- ellipsoidal	4.0–7.5 × 3.5–4.5	obovate-rhombic to oblong-obovate	2.0–3.0 × 1.8–2.0	rhombic	rounded; reflexed	sunken; recurved distally	Fu *et al.*^5^
*P. wangii*	oblong-ellipsoidal or cylindrical-ovoid	4.5–9.0 × 2.0–4.5	sub-obovate	2.0–3.0 × 1.5–2.0	transversely rhombic	thin; slightly incurved	sunken; not swollen	Fu *et al.*^5^
*P. kwangtungensis*	cylindrical-oblong or cylindrical-ovoid	3.0–17.0 × 1.5–7.0	cuneate-obovate	2.5–3.5 × 1.5–2.3	rhombic	thin; slightly recurved	flat; straight or slightly incurved	Fu *et al.*^5^
*P. morrisonicola*	conical-ellipsoidal or ovoid-ellipsoidal	7.0–11.0 × 5.0–7.0	cuneate-elliptical	3.0–3.5 × 1.5–2.0	broadly rhombic	obtuse thickened; recurved	recurved	Fu *et al.*^5^
*P. chiapensis*	sub-cylindrical	6.0–25.0 (length)	cuneate-elliptical	ca.3 × 1.0–1.5	broadly rhombic	thin, concave; not reflexed	slightly incurved	Earle^3^
*P. peuce*	cylindrical, straight to slightly curved	5.0–20.0 (length)	cuneate-elliptical	2.0 (width)	broadly rhombic	thin, rounded; slightly incurved	flat or slightly incurved	Earle^3^
